# The Junior Students’ Internet Literacy Scale: Measure Development and Validation

**DOI:** 10.3390/ijerph181910120

**Published:** 2021-09-26

**Authors:** Yinghui Huang, Hui Liu, Weijun Wang, Rouchun Dong, Yun Tang

**Affiliations:** Key Laboratory of Adolescent Cyberpsychology and Behavior, Ministry of Education, Central China Normal University, Wuhan 430079, China; yhhuang@ccnu.edu.cn (Y.H.); huiliu931031@gmail.com (H.L.); dongrouchun@mails.ccnu.edu.cn (R.D.); tangyun@mail.ccnu.edu.cn (Y.T.)

**Keywords:** Internet literacy, Internet literacy scale, Internet addiction, junior students

## Abstract

Despite the great attention paid to Internet literacy research, little has been done to overcome the problems stemming from the heterogeneity of Internet literacy nomenclature and the use of non-standardized measurement tools, especially for adolescents in developing countries. Considering junior students are the high-risk groups of Internet addiction and have wide access to the Internet, the aim of this study is to develop a new scale to assess Chinese junior students’ Internet literacy (JIL). In the psychometric study (*n* = 1099 junior students), an 18-item scale was developed using the exploratory and confirmatory factor analyses, which includes five subscales: knowledge and skills for the Internet (KSI), Internet self-management (ISM), awareness and cognition of Internet (ACI), Internet interactions (II), and autonomous learning on the Internet (ALI). Evidence of internal reliability, test-retest reliability, and construct validity provided good psychometric support for the measure. Criterion-related validity of the measures was demonstrated by examining its anticipated theoretical relations to two hypotheses: (1) High JIL level alleviates the adverse effects of an individual’s Internet addiction degree, while pathological use for interacting with others on the Internet exacerbates the adverse effects; (2) an individual’s degree of Internet use self-efficacy is positively associated with JIL level. It is envisaged that the JIL Scale will help facilitate unified research in the field.

## 1. Introduction

As an emergency response to the COVID-19 epidemic, the Chinese government launched the world’s largest online education practice. As of December 2020, the number of netizens in China reached 989 million, of which the netizens aged 0–19 accounted for 16.6% of the total netizens [1]. Internet and digital technology have been shaping the lives of children and adolescents, growing up with it, and providing unlimited learning and social opportunities [2]. At the same time, it puts children’s safety, privacy, physical and mental health at huge risk, leading to online bullying, Internet dependence, “screen addiction" and “problematic Internet use” [3]. Psychological support and behavioral therapy for elementary and junior students in cyberspace have become an important part of traditional education [4,5,6,7].

The concept of Internet literacy was put forward firstly by McClure, which consists of Internet knowledge and Internet skills [8]. With the continuous development of ICT (information and communication technology), the Association of College and Research Libraries (ACRL) proposed information literacy standards for student learning, which include the ability to access, evaluate, understand and use information effectively and efficiently [9]. Livingstone pointed out that generalized Internet literacy mainly referred to people’s ability to approach, analyze, evaluate and produce Internet media content [10]. Increasing numbers of studies have provided insights into the understanding of the psychosocial and behavioral effects of Internet literacy [11,12,13,14]. The relationships between junior students’ Internet literacy (JIL) and problematic Internet use [15,16], academic achievements, Internet addiction [17,18,19,20], Internet self-efficacy [21,22,23], and parenting styles [13,17] has been widely confirmed. For example, many studies demonstrated that the competencies of the individual in using the Internet may be preventive for the development of pathological use of social networking sites or other Internet activities [17,18,20]. Additionally, Hatlevik, Throndsen, Loi, and Gudmundsdottir reported obvious relationships between ICT self-efficacy and computer and information literacy [21].

Extant measures of Internet literacy tend to be study-specific and have their structure and emphasis. Most researches have focused on college students or individuals, and little attention has been paid to the junior students [13,19,24,25,26,27,28,29,30]. The general purpose of the current study is to develop and validate a new measure of the JIL Scale, assessing the Chinese junior students’ Internet literacy level. This overarching goal was accomplished via four specific tasks. First, we identified potential items to represent each of the eight subtypes of the JIL Scale that have predominated in the Internet literacy-related literature. Second, we used factor analysis to affirm the eight-factor structure of the new measure and demonstrate that a five-factor solution can optimally fit the data. Third, we demonstrated Internal and test-retest reliability and constructed validity. Fourth, we demonstrated the criterion-related validity of the measure by examining its anticipated theoretical relations vis-à-vis two hypotheses: (1) JIL will alleviate the adverse effects of an individual’s Internet addiction degree, while pathological use for interacting with others on the Internet will exacerbate the adverse effects of Internet addiction. (2) An individual’s degree of Internet use self-efficacy will be positively associated with JIL level.

## 2. Present Study

To explore the internal meaning and characterize the structure of JIL, we began by conducting a literature review related to Internet literacy and its measures. This study used "SU = Network Literacy" to retrieve 987 Chinese papers, including 817 journal papers and 120 master theses and doctoral dissertations from the National Knowledge Infrastructure of China. We obtained English papers from the core data set of Web of Science by query such as “TS = ((Internet literacy) OR (network literacy) OR (cyber literacy) OR (online literacy) OR (net literacy) OR (cyber wellness))” as the search formula, and 1653 English papers were obtained (search time is May 2019 30th). Then, we sorted out 19 representative papers on Internet literacy (including conceptual connotation, evaluation indicators, scales, etc.) according to the reputation of the journal, author, and research institute.

We further examined the number and nature of identified Internet literacy subtypes from the above 19 representative papers. After collapsing across conceptually similar subtypes and subtypes with poor discriminant validity, we identified 20 different subtypes of Internet literacy using a top-down approach. In Table 1, we present a modified stem-and-leaf plot of these results, in which Internet literacy subtype names are the stems and numeric representations of the publications are the leaves. 

As shown in Table 1, the skill-based Internet literacy was measured as a composite scale of proficiency for specific online activities in Lee and Chae’s study [13], including competence related to accessing and evaluating information and competence related to online communication and interaction. Stodt, Wegmann, and Brand developed a 24-item Internet Literacy Questionnaire (ILQ), including four dimensions, which are technical expertise, reflection, and critical analysis, production and interaction, and self-regulation [19]. Based on the 24-item questionnaire, Stodt et al. further developed a refined version of ILQ, which includes 18 items on the four above-mentioned dimensions [29]. Bauer and Ahooei reviewed the existing notions and classifications of Internet literacy and used the grounded theory method to conclude a rearticulated version of Internet literacy [28]. The new classification was established based on the three main components, including responsibility, productivity, and interactivity. In addition, Wu, Na, and Li designed an Internet information literacy scale for college students, including four dimensions such as information awareness, information skills, information application and creation, and information ethics and security [27]. Furthermore, B. M. Li compiled the children’s Internet literacy questionnaire from four dimensions, which are knowledge and awareness of the Internet, emotional experience and value orientation about the Internet, decision-making and judgment skills, and online behaviors norm [26]. Q. Li developed an Internet literacy questionnaire for junior students in one city of China, which includes five dimensions such as cognition and technology of the Internet, ability to select online information, ability to extend self-consciousness by the Internet, self-discipline and cybersecurity awareness [25]. Another seven-dimensional Internet literacy scale for college students was developed by Shapiro and Hughes, which comprised tool literacy, resource literacy, social-structural literacy, research literacy, publishing literacy, emergent technology literacy, and critical literacy [24]. Then Wu revised it and made a Chinese version [30].

Based on the 20 original subtypes of Internet literacy showed in Table 1, 8 subtypes of Internet literacy were summarized. The corresponding relationships between original subtypes and new subtypes of Internet literacy are shown in Table 2. Therefore, through literature reviews, the definition of Internet literacy for junior students in this study is the comprehensive ability of adaptation and development on the Internet, that is, their abilities to properly use, adapt well, develop healthily, and explore innovatively in the Internet environment.

## 3. Methods

### 3.1. Junior Students’ Internet Literacy Scale (JIL Scale) Development

To obtain items representing all necessary aspects of Internet literacy for junior students, we derived and adapted items from three sources: literature related to Internet literacy, pre-existent measures of Internet literacy, and interviews on Internet literacy issues for school students. In the present study, we extracted 20 different subtypes of Internet literacy from literature and extant measures of Internet literacy using a top-down approach and summarized the 20 subtypes to 8 subtypes, which can reflect broad Internet literacy meaning (see Table 2). Then from the interviews, we recruited 48 interviewees, which include junior high school teachers, academics, and social professionals engaged in related research and educational practices. Combined with the 8 subtypes summarized by related literature and extant measures of Internet literacy, we built our items pool from interview materials using a bottom-up approach [39]. Every interviewee was asked about the meaning of junior students’ Internet literacy and how to evaluate it (see Appendix A).

After sorting out relevant literature, pre-existent measures of Internet literacy, and interview materials, we extracted 55 initial JIL Scale items, which could reflect junior students’ Internet literacy. The initial scale comprises eight dimensions of knowledge and skills for Internet (KSI), Internet self-management (ISM), Internet interactions (II), autonomous learning on the Internet (ALI), self-protection (SP), cognition of Internet (CI), norms of Internet (NI), and value identity (VI). To examine the content validity of the scale, we invited a psychologist and 3 Ph.D. students in related fields to evaluate the division and naming of the scale dimensions to ensure that the dimensional setting is rigorous and clear; and 10 junior students were invited to understand the content of the scale items to ensure those language expressions are clear and accurate.

After two preliminary tests in Sample 1 and Sample 2 (see Table 3), the 37 JIL Scale items were obtained, which were rated on a 5-point Likert scale: 1 (‘‘strongly disagree’’), 2 (‘‘somewhat disagree’’), 3 (‘‘neutral’’), 4 (‘‘somewhat agree’’), and 5 (‘‘strongly agree’’). Except for ISM 1, ISM 2, and ISM 3 items, all items are scored in the forward direction. Participants chose the corresponding answers based on their real situation. The score of the subscale is the sum of the scores of the subscale items, and the total score of the scale is the sum of all items. It is worth noting that the JIL Scale developed does not measure a participants’ true Internet literacy level but rather their competence beliefs.

### 3.2. Participants

Before determining the initial 55 JIL Scale items in the scale, we recruited 48 participants to conduct interviews around the internal meaning and framework of JIL. These participants consist of 15 junior high school teachers, 18 academics engaged in related research, and 14 social professionals engaged in educational practices. 

To ensure a high degree of generalizability, we collected data from three different cities in China. After two pretests in Sample 1 (*n* = 171) and Sample 2 (*n* = 897), we refined the initial 55 JIL Scale items to the 37 JIL Scale items. We conducted all analyses on the 37 JIL Scale items for Sample 3 (*n* = 1099) and obtained the formal JIL Scale (18 items). Finally, we utilized Sample 4 (*n* = 120) to verify the test-retest reliability of the formal JIL Scale (18 items).

Table 3 summarizes all relevant socio-demographic information collected from all samples. The total samples comprising 2173 junior students were recruited to take part in multipart data acquisition by filling out paper questionnaires. The offline data collection methodology was chosen because of the difficulties in finding samples of junior students accurately on the Internet. In the process of refining the initial 55 JIL Scale items, we recruited 171 junior students (Sample 1) from City 1 (88 females, 82 males, 1 unknown; age: M = 13.27, SD = 0.56, range: 12–15 years) and 897 junior students (Sample 2) from City 2 (452 females, 444 males, 1 unknown; age: M = 13.64, SD = 0.82, range: 12–17 years) to obtain the 37 JIL Scale items. Then, we recruited 1105 junior students from City 3 to investigate the 37 JIL Scale items’ factor structure. A total of 6 out of 1105 (0.5%) questionnaires were excluded from the final analyses due to the incomplete data or nonvariance of response. Thus, the remaining 1099 questionnaires (Sample 3, 452 females, 640 males, 7 unknown; age: M = 12.36, SD = 0.48, range: 12–13 years) were conducted using factor analysis and obtained the formal JIL Scale (18 items). Finally, we recruited 120 junior students (Sample 4) from City 1 (57 females, 63 males; age: M = 12.52, SD = 0.65, range: 11–14 years) to repeat the test in one month to analyze the test-retest reliability of the formal JIL Scale (18 items).

### 3.3. Measures

In addition to the 37-item version of the JIL Scale (described above), we administered measures of Internet addiction, Internet use self-efficacy, and family affluence.

#### 3.3.1. Internet Addiction Test (IAT)

Our measure of Internet addiction degree was the Internet Addiction Test [40], which comprises 20 items rated on a 5-point Likert scale: 1 (‘‘Not at all’’), 2 (‘‘Rarely’’), 3 (‘‘Occasionally’’), 4 (‘‘Often’’), and 5 (‘‘Always’’). The scores are obtained by summing the items, and total scores can range from 20 to 100, and higher than 80 means Internet addiction. In the present study, the Cronbach’s alpha for the IAT was 0.896. This measure was used to examine the JIL Scale concurrent validity should a significant negative correlation be observed between the two measures.

#### 3.3.2. Internet Use Self-Efficacy Questionnaire

The Internet Use Self-efficacy Questionnaire was developed by Eastin and LaRose [41] and then revised by Luo et al. [42]. This instrument aims to assess individuals’ confidence in their ability to use the Internet to produce overall attainments. The Questionnaire comprises 9 items rated on a 4-point Likert scale: 1 (‘‘Strongly Disagree’’), 2 (‘‘Disagree’’), 3 (‘‘Agree’’), and 4 (‘‘Strongly Agree’’). The scores are obtained by summing the items and total scores can range from 9 to 36, with higher scores being indicative of higher degrees of Internet use self-efficacy. In the present study, the Cronbach’s alpha for the questionnaire was 0.905. This measure was used to examine the JIL Scale concurrent validity should a significant positive correlation be observed between the two measures.

#### 3.3.3. Family Affluence Scale (FAS)

The FAS was developed by Currie et al. [43], which included 4 questions relating to the number of cars, bedroom occupancy, family holidays, and family computers to assess the wealth of the family. This scale has good reliability and validity.

### 3.4. Statistical Analysis

Statistical analysis comprised of (i) descriptive statistics of the main sample’s characteristics and (ii) a psychometric study of the JIL Scale. These latter analyses encompassed an exploratory factor analysis (EFA), confirmatory factor analysis (CFA), assessment of the validity and reliability. 

For the CFA goodness of fit, a *p*-value of chi-square smaller than 0.05 for the test of close fit was considered. Additionally, other fit indices included Comparative Fit Index (CFI), Tucker–Lewis Fit Index (TLI), Root Mean Square Residual (SRMR). For both CFI and TLI, values greater than 0.80 were considered good whereas values above 0.95 were considered optimal. Moreover, an RMSEA value smaller than 0.08 expresses an acceptable fit, whereas an optimal fit is expressed by a value close to 0.06 [44,45,46].

To carry out the analyses, MPLUS 7 (Muthén & Muthén, Los Angeles, CA, USA) was used for the CFA, JASP 0.11.1.0 (JASP Team, Amsterdam, Netherlands)was used for parallel analysis, and IBM SPSS Statistics Version 22 (IBM Corp, Armonk, NY, USA) for the remaining analyses. All statistical tests adopted a significance level of 0.05.

## 4. Results

### 4.1. Preliminary Analyses

After pretesting twice, we selected 1099 junior students (Sample 3) from City 3 to investigate 37 JIL Scale items’ factor structure. Furthermore, we examined Sample 3 concerning their parents’ education level and their family affluence level. As Table 4 shows that, the education level of fathers and mothers at bachelor degree or higher accounted for 79.07% and 70.34%, respectively, in this sample. A total of 90.7% of households have at least one car, 90.1% of students have their bedroom, and during the past 12 months, 91.7% of students have traveled away on holiday with their family at least one time. Moreover, 98.6% of households have at least one computer, and 67.8% of households have more than two computers.

### 4.2. Exploratory Factor Analysis (EFA)

Before investigating the JIL Scale factor structure (i.e., EFA and CFA), the whole sample 3 was randomly split into two samples (Sample 3a (*n* = 550) and Sample 3b (*n* = 549)). Therefore, an EFA using the principal component analysis method with oblique rotation on the 37 JIL Scale items was performed in Sample 3a (*n* = 550) to examine its factorial structure. The number of components to be extracted was determined through an examination of the scree plot [47] in combination with the conventional Kaiser criterion [48] (i.e., all factors with eigenvalues greater than one). In addition, a parallel analysis [49] was conducted as an additional method of determining the number of factors to be extracted for the EFA and supported that five factors were the appropriate number. Furthermore, interpretation of the factors was guided by the examination of the standardized regression coefficients. Items with relatively low individual loadings (<0.40), crossloadings of 0.30 or higher, content redundant or content inconsistent with the other items grouped in its factor were removed. Then, an EFA was conducted again on the remaining 18 items of the JIL Scale to make sure that five factors were the appropriate number.

The appropriateness for conducting the EFA was confirmed by the Kaiser–Meyer–Olkin Measure of Sampling Adequacy (KMO = 0.814) and Bartlett’s Test of Sphericity ($\chi^{2}$(550) = 2193.055, *p* < 0.05) results [46,50]. The analysis revealed the five factors explaining 55.96% of the total variance of the construct and was extracted after 25 iterations (see Table 5).

Among them, Factor 1 has four items, reflecting Knowledge and Skills for Internet (KSI); Factor 2 has three items, reflecting Internet Self-management (ISM); Factor 3 has five items, comprising Self-protection (SP), Cognition of Internet (CI), Norms of Internet (NI) and Value Identity (VI), so it is renamed Awareness and Cognition of Internet (ACI); Factor 4 has three items, reflecting Internet Interactions (II); and Factor 5 has three items, reflecting Autonomous Learning on the Internet (ALI). The 5-factor structure is the same as the previous 8-factor structure, except for Awareness and Cognition of Internet (ACI) merging previous Self-protection (SP), Cognition of Internet (CI), Norms of Internet (NI), and Value Identity (VI), which is to assess the extent of their Internet-related awareness and cognition. The 18 JIL Scale items are the same as for the final version of the measure (see Appendix B).

### 4.3. Confirmatory Factor Analysis (CFA)

To confirm the five-factor solution found of the 18 JIL Scale items obtained in the EFA, a CFA with the maximum-likelihood method was performed on Sample 3b (*n* = 549) using the 18 JIL Scale items to corroborate the factor structure found previously. 

For the CFA goodness of fit, the analysis of the five factors model provided an acceptable model fit for the JIL Scale. More specifically, $\chi^{2}/df=13.75$, CFI = 0.903, TLI = 0.881, RMSEA = 0.053, SRMR = 0.049. As shown in Table 6, all factor loadings were statistically significant and within the conventionally acceptable threshold of >0.40 [51].

### 4.4. Reliability Analysis in Sample 3b and 4

Internal Reliability: Internal reliability was examined for the formal JIL Scale (18 items) and its subscale scores in sample 3b. Cronbach’s alpha was employed to estimate the internal consistency of the dimensions validated by the CFA [52]. It provided an overall measure of the interrelatedness among the items comprising each dimension. Values of Cronbach’s alpha greater than 0.6 were considered to reflect an acceptable level of reliability [53]. In light of the aforementioned assumptions, the value for Cronbach’s alpha of total scale is 0.74. And the values for Cronbach’s alpha of KSI, ISM, ACI, II, and ALI are 0.65, 0.68, 0.60, 0.70, and 0.62, respectively.

Test-Retest Reliability: In addition, 120 junior students (Sample 4) were selected to repeat the test in one month to analyze the test-retest reliability. Test-retest reliability measures the consistency of results when the participants repeat the same test on the same sample at a different point in time. Cicchetti defined test-retest reliability of 0.4 to 0.59 as fair, 0.60 to 0.74 as good, and above 0.75 as excellent [54]. In light of the aforementioned assumptions, the test-retest reliability of the total scale is 0.74, and the values of KSI, ISM, ACI, II, and ALI are 0.68, 0.72, 0.62, 0.74, and 0.61, respectively.

### 4.5. Validity Analysis in Sample 3b

Correlations: We examined correlations of the formal JIL Scale (18 items) and its subscales for evidence of construct validity (see Table 7). Firstly, the results of the confirmatory factor analysis have initially shown that the scale is reasonably structured. Secondly, in this study, the correlation between the scores of the subscales, and between the scores of the total scale and subscales was analyzed to examine the construct validity of the formal JIL Scale (18 items). The results are shown in Table 7. The correlations between subscales were between 0.136 and 0.452, and the correlations between the subscales and the total score were between 0.435 and 0.727. As for the ISM subscale, it has significant positive correlations with the KSI, ACI, and ALI subscale, while it has significant negative correlations with the II subscale.

We also examined the relations of the formal JIL Scale (18 items) and its subscales with the IAT (Internet Addiction Test) and Internet Use Self-efficacy Questionnaire for evidence of criterion-related validity. As Table 7 shows, there are significant negative correlations between the JIL Scale and its subscales with the IAT (except for the II subscale, it has significant positive correlations with the IAT scale), which verify the hypothesis: (1) high JIL level will alleviate the adverse effects of individual’s Internet addiction degree, while pathological use for interacting with others on the Internet will exacerbate the adverse effects of Internet addiction. In addition, the significantly positive correlations between JIL Scale and its subscales with the Internet Use Self-efficacy Questionnaire (except for the ISM subscale, it has not significantly correlations with the Internet Use Self-efficacy Questionnaire) verify the hypothesis: (2) individuals’ degree of Internet use self-efficacy will be positively associated with JIL level.

## 5. Discussion and Conclusions

The purpose of this study was to develop a new scale to assess Chinese junior students’ Internet literacy based on the summary of related literature and the results of interviews. To achieve this goal, two steps were taken. Firstly, 55 potential JIL Scale items were derived from related literature and interviews, and subsequently, the 37 JIL Scale items were obtained after refining twice for Sample 1 (*n* = 171) and Sample 2 (*n* = 897), respectively. Secondly, the 37 JIL Scale items were subject to in-depth psychometric examination to ascertain that they have appropriately reflected the concept of JIL. The results demonstrated the five-factor solution for JIL using the 18 items of the JIL Scale. This structure emerged in the EFA and was later confirmed by the CFA results that provided fit indices that confirmed the viability of the proposed five-factor solution as the model optimally fitted the data.

Existing research on Internet literacy mostly focuses on college students or individuals, while there is little research on junior students’ Internet literacy. Consequently, the present study represents a new contribution to the Internet literacy literature by providing a new and valid psychometric tool for assessing Chinese junior students’ Internet literacy. Therefore, future research should investigate whether the five-factor solution applies to other samples in different contexts and populations. If the present JIL Scale can be replicated in future studies, it will potentially help develop a standardized tool to measure Chinese junior students’ Internet literacy levels. In addition, future research can develop more comprehensive standardized measurement tools for people of different ages/countries (or regions).

In terms of the scale’s reliability and validity, the JIL Scale appeared to be a valid and reliable measure for assessing JIL. The Cronbach’s alpha coefficients of the JIL Scale and its subscales were ranged from 0.60 to 0.74, and the test-retest reliabilities were between 0.61 and 0.74. Cronbach’s alpha was employed to estimate the internal consistency of the dimensions validated by the CFA [52]. It provided an overall measure of the interrelatedness among the items comprising each dimension. Values of Cronbach’s alpha greater than 0.6 were considered to reflect an acceptable level of reliability [53]. Test-retest reliability measures the consistency of results when the participants repeat the same test on the same sample at a different point in time. Cicchetti defined test-retest reliability 0.4 to 0.59 as fair, 0.60 to 0.74 as good, and above 0.75 as excellent [54]. The JIL Scale we developed comprised broad dimensions, not only skills and abilities but also awareness. We think this is the main reason for the relatively low reliability.

Moreover, the correlations between subscales were between 0.136 and 0.452, and the correlations between the JIL Scale and its subscales were between 0.435 and 0.727. The correlation coefficient of the JIL Scale and its subscales is larger than that of each other, which shows that all subscales have good independence and reflect the characteristics to be measured. As for the Internet Self-management (ISM) subscale, it has significant positive correlations with the Knowledge and Skills for Internet (KSI), Awareness and Cognition of Internet (ACI), and Autonomous Learning on the Internet (ALI) subscales, while it has significant negative correlations with the Internet Interaction (II) subscale.

The Internet Self-management (ISM) subscale mainly assesses the students’ self-control ability and whether they can allocate their time properly when surfing the Internet. Junior students prefer online chat (73.1%) and using social networking sites (45.8%) to other minors. At the same time, the proportion of online games with social elements is 64.7% [2]. From this, we can see that Internet interaction plays an important role in junior students’ online activities. Some studies also show that the individual’s addiction to online social interaction is related to their lack of self-management ability [20,55,56]. Therefore, in our study, the significant negative correlation between the Internet Self-management (ISM) subscale and Internet Interaction (II) subscale also confirms this conclusion.

For the significant positive correlations between Internet Self-management (ISM) and Knowledge and Skills for Internet (KSI), Awareness and Cognition of Internet (ACI) subscale, and Autonomous Learning on the Internet (ALI) subscales, preliminary research has provided evidence consistent with the outcomes. For example, Valcke, Bonte, De Wever, and Rots found that parents of high education and urban families will use more strategies to explain the rules of Internet use, to communicate with their children, to support and guide their children to use the Internet safely and reasonably [57]. In addition, by analyzing the interaction between parents and their children in the use of multimedia and their children’s behaviors related to online activities; Symons et al. and Soh et al. found that parents recommending useful websites to their children and accompanying their children online can promote their children to actively participate in online education activities such as online learning and reduce their children’s online risky behavior [58,59]. For the junior students in Sample 3, Table 4 shows that their parents who are highly educated and have rich experience in using the Internet can regulate children’s online behavior. Hence, their Internet self-management level is positively related to their knowledge and skills for Internet, the awareness and cognition of Internet, and the autonomous learning on the Internet.

As for the correlations of the JIL Scale and its subscales with the IAT (Internet Addiction Test) and Internet Use Self-efficacy Questionnaire, there are significant negative correlations between the JIL Scale and its subscales with the IAT (except for Internet Interaction (II) subscale, it has significant positive correlations with the IAT scale), while there are significant positive correlations between the JIL Scale and its subscales and the Internet Use Self-efficacy Questionnaire (except for the Internet Self-management (ISM) subscale, which does not have significant correlations with the Internet Use Self-efficacy Questionnaire). Many studies have also come to the same conclusion. Chou and Chou, Stodt, Wegmann, and Brand, Wegmann, Stodt, and Brand and Leung and Lee found that severe Internet addiction symptoms can be associated with lower self-regulation and pathological use of Internet activities [17,18,19,20,60]. Furthermore, Leung and Lee discovered that the more savvy adolescents are with technology (especially in SNS and online games), the less knowledge they had of locating, browsing, and accessing information online and understanding Internet-related awareness, the more they will exhibit addiction symptoms, especially in losing control of the amount of time spent on the Internet [18]. Thus, the adolescents who are addicted to the Internet tend to spend more time participating in Internet activities, especially in SNS and online games, but they lack other Internet-related knowledge and skills and lack Interent awareness and cognition, not to mention the ability to conduct online learning. 

For the positive correlations between Internet Use Self-efficacy and Knowledge and Skills for Internet (KSI), Awareness and Cognition of Internet (ACI), Internet Interaction (II), and Autonomous Learning on the Internet (ALI), Bandura proposed that self-efficacy is the individuals’ perception of and confidence in their abilities to perform a behavior successfully [61]. Individuals’ self-efficacy levels influence their ability to acquire skills, SNS use, and willingness to continue in a course of action [62]. Hatlevik et al. show that autonomous learning and having experience with ICT are important for students’ confidence in using technology and their beliefs about what they can accomplish using ICT [21]. According to motivation theory [63], efficacious students are not afraid of coping with challenging tasks. They also use effective cognitive and metacognitive strategies when faced with obstacles or difficult situations; they use effective procedures, monitor, and evaluate their progress, and adjust strategies if needed. Therefore, students who have higher degrees of self-efficacy in using the Internet may have more confidence and motivation to obtain Internet-related knowledge and skills, develop the awareness and cognition of Internet, interact with others, and learn using the Internet.

The present study is not without limitations. Firstly, the use of convenience samples, despite being common practice across various domains of the psychological literature, is not without its problems. In the present study, despite our participants coming from three different cities in China, the majority of our research participants are of Han nationality, which lacks the ethnic minority participants. Hence, these findings should be cautiously interpreted in terms of their generalizability. Future studies should aim to replicate the present findings using more generalized samples. Secondly, although we used Sample 4 (*n* = 120) to test the test-retest reliability of the JIL Scale, there is still a lack of measurement invariance tests for different samples, age, gender, and other important variables. Future studies can utilize measurement invariance tests to perfect this research. Third, we did not verify the scale using observer-rating information from parents, teachers, and peers and ecological data of online behavior. We will further verify the validity of the scale by using observer-rating information and ecological data.

Overall, the findings of the present study lend empirical support for the concept of JIL as suggested by the references and interviews while also supporting the viability of further study of this phenomenon. Moreover, the current findings suggested that the JIL Scale can cater to the generalized need for a standardized and psychometrically sound tool for assessing junior students’ Internet literacy. Consequently, it is envisaged that this new tool will help facilitate research in the field by providing a concise, valid and reliable instrument for measuring JIL.

## Figures and Tables

**Table 1:** Stem-and-leaf plot of literature supporting different subtypes of Internet literacy.

Subtypes	Numeric Code for Supporting Citations																		
Internet-related knowledge	01	02	03	04	05	06	07	08	09	10	11	12	13	14	15	16	17	18	19
Internet-related skills	01	02	03	04	05	06	07	08	09	10	11	12	13	14	15	16	17	18	19
Tell untrustworthy content	03	06	07	08	09	10	11	12	13	14	15	16	17	18	19				
Cognize and access information resources	03	05	06	07	08	09	10	11	12	13	16	17	19						
Product information	03	04	05	07	08	09	10	13	15	17	18	19							
Communicate and interact with others on the Internet	03	08	09	10	11	14	15	17	18	19									
Utilize the Internet for learning and improve themselves	02	04	05	06	07	09	11	12	17										
Self-management	04	06	07	12	15	18	19												
Process information	04	05	08	13	14	16	17												
Information communication	03	04	07	08	09	17													
Internet security awareness	04	06	08	10	12														
Reflective ability	11	15	16	17	18														
Internet morality	06	10	11																
Self-regulation	11	15	18																
Emotional experience and values	05	11																	
Inquiry ability	11	17																	
Information immune	07																		
Internet behavior habits	11																		
Internet responsibility consciousness	19																		
Internet legal literacy	11																		

Note: 01: McClure, 1994 ([8]); 02: ACRL, 2000 ([9]); 03: Savolainen, 2002 ([31]); 04: Chen and Yang, 2004 ([32]); 05: Z. Y. Li, 2005 ([33]); 06: Bei, 2006 ([34]); 07: Huang, 2007 ([35]);. 08: Livingstone, 2008 ([11]); 09: Ngulube, Shezi, and Leach, 2009 ([36]); 10: Wu, Na, and Li, 2009 ([27]); 11: B. M. Li, 2012 ([26]); 12: Q. Li, 2012 ([25]); 13: Lee and Chae, 2012 ([13]); 14: Rheingold, 2012 ([37]); 15: Stodt, Wegmann, and Brand, 2016 ([19]); 16: Kim and Yang, 2016 ([38]); 17: Wu, 2017 ([30]); 18: Stodt et al., 2018 ([29]); 19: Bauer and Ahooei, 2018 ([28]).

**Table 2:** The corresponding relationships between original subtypes and new subtypes of Internet literacy.

Original 20 Subtypes	Summarized 8 Subtypes	Definition
∙ Internet-related knowledge ∙ Access information resources ∙ Product information ∙ Internet-related skills ∙ Process information	Knowledge and Skills for Internet (KSI)	To assess the level of their basic Internet-related knowledge and basic Internet-related skills.
∙ Self-regulation ∙ Self-management ∙ Internet behavior habits	Internet Self-management (ISM)	To assess their self-control ability and to what extent they can allocate their time properly when surfing the Internet.
∙ Information communication ∙ Communicate and interact with others on the Internet	Internet Interactions (II)	To assess to what extent they can communicate and interact with others on the Internet.
∙ Utilize the Internet for learning and improve themselves ∙ Reflective ability ∙ Inquiry ability	Autonomous Learning on the Internet (ALI)	To assess to what extent they can study spontaneously on the Internet.
∙ Tell untrustworthy content ∙ Information immune ∙ Internet security awareness	Self-Protection (SP)	To assess to what extent they can tell or be immune to untrustworthy and harmful information.
∙ Cognize information resources	Cognition of Internet (CI)	To assess to what extent they can cognize the importance and two sides of Internet information resources.
∙ Internet legal literacy ∙ Internet morality ∙ Internet responsibility consciousness	Norms of Internet (NI)	To assess to what extent they can abide by moral and legal norms on the Internet and have a sense of responsibility.
∙ Emotional experience and values	Value Identity (VI)	To assess to what extent they can respect different Internet cultures around the world.

Note: The original subtype “cognize and access information resources” was divided into “cognize information resources” and “access information resources”.

**Table 3:** Socio-demographic characteristics of the samples.

Samples	Cities	*n*	Gender (Male, *n*, %)	Age, Years; Mean (SD)
Sample 1	City 1	171	82 (48.0)	13.27 (0.56)
Sample 2	City 2	897	444 (49.5)	13.64 (0.82)
Sample 3	City 3	1099	640 (58.2)	12.36 (0.48)
Sample 4	City 1	120	63 (52.5)	12.52 (0.65)

**Table 4:** The statistics of parents’ education level and family affluence level for Sample 3 (*n* = 1099).

Education Level (*n*, %)		
	**Father**	**Mother**
High school or below	230 (21)	326 (29.7)
Bachelor degree	662 (60.2)	643 (58.5)
Graduate degree	207 (18.8)	130 (11.8)
**FAS (*n*, %)**		
1. Car: does your family own a car, van, or truck?		
No	102 (9.3)	
Yes, one	618 (56.2)	
Yes, two or more	379 (34.5)	
2. Own bedroom: do you have your bedroom for yourself?		
No	109 (9.9)	
Yes	990 (90.1)	
3. Holidays: during the past 12 months, how many times did you travel away on holiday with your family?		
Not at all	91 (8.3)	
Once	209 (19.0)	
Twice	292 (26.6)	
More than twice	507 (46.1)	
4. Computers: how many computers does your family own?		
None	15 (1.4)	
One	116 (10.5)	
Two	223 (20.3)	
More than two	745 (67.8)	

**Table 5:** Summary of the results from the EFA on the 18 JIL Scale items for Sample 3a (*n* = 550).

Items	Contents	Factor 1	Factor 2	Factor 3	Factor 4	Factor 5	Communalities
KSI1	I can use some Internet tools like office software and search engine.	0.694					0.499
KSI2	I can use Internet resources creatively, such as searching for picture materials to make PowerPoint.	0.687					0.497
KSI3	I can use what I have learned in the information technology class when I go online.	0.655					0.499
KSI4	I can express my opinions and ideas through various media such as text, sound, images, etc.	0.621					0.562
ISM1	I can’t control how much time I spend on the Internet.		0.804				0.651
ISM2	When I study online, I am easily attracted by other irrelevant information.		0.726				0.556
ISM3	I don’t have a plan when I’m surfing the Internet.		0.702				0.531
ACI1	Keep a clear head and discern harmful information while surfing the Internet.			−0.834			0.631
ACI2	I realize that Internet has two sides.			−0.760			0.602
ACI3	If someone disagrees with me online, I will communicate with him sensibly instead of scolding him.			−0.530			0.465
ACI4	We should respect different Internet cultures in the world.			−0.440			0.396
ACI5	I can immune to bad information while online.			−0.397			0.345
II1	I am good at using the Internet to expand my relationships.				0.854		0.749
II2	I can make new friends through the Internet.				0.800		0.693
II3	I can show myself through social media such as WeChat, QQ, Douyin, and so on.				0.643		0.501
ALI1	I can find my favorite learning method online.					−0.850	0.700
ALI2	I can access information via the web and sort out useful information to complete the learning tasks.					−0.709	0.559
ALI3	I can use the Internet to improve myself, such as learning meaningful knowledge or skills.					−0.706	0.637
	Eigenvalues	4.010	2.497	1.439	1.123	1.005	
	% of the Variance	22.28	13.87	7.99	6.24	5.58	

**Table 6:** Summary of the results from the CFA on the18 JIL Scale items for Sample 3b (*n* = 549).

Items	Factor 1	Items	Factor 2	Items	Factor 3	Items	Factor 4	Items	Factor 5
KSI4	0.629	ISM1	0.768	ACI1	0.585	II1	0.827	ALI3	0.720
KSI2	0.606	ISM3	0.613	ACI5	0.565	II2	0.743	ALI2	0.607
KSI3	0.532	ISM2	0.558	ACI2	0.530	II3	0.470	ALI1	0.526
KSI1	0.511			ACI4	0.406				
				ACI3	0.402				

**Table 7:** Correlations for all study variables for Sample 3b.

	JIL Scale	KSI	ISM	ACI	II	ALI
KSI	0.727 **					
ISM	0.435 **	0.139 **				
ACI	0.672 **	0.380 **	0.244 **			
II	0.564 **	0.302 **	−0.195 **	0.150 **		
ALI	0.686 **	0.401 **	0.136 **	0.452 **	0.306 **	
IAT scale	−0.248 **	−0.195 **	−0.501 **	−0.245 **	0.237 **	−0.109 *
Internet Use Self-efficacy Questionnaire	0.362 **	0.398 **	0.054	0.152 **	0.220 **	0.288 **

Note. *n* = 549. * *p* < 0.05, ** *p* < 0.01.

## Appendix A

The interview outline consists of 4 questions:

1. What do you think of Internet literacy?
2. In what respect do you think the level of Internet literacy of junior students is reflected?
3. From which dimensions do you think we should evaluate the level of Internet literacy of junior students?
4. What do you think of the characteristics of Internet literacy of junior students?

## Appendix B

Hello students! Our lives cannot be separated from the Internet. We are interested in how you use the Internet in daily life and how you think about some Internet related activities. Now, please rate how much the following items you agree with. Use the following scale:

1 = Strongly disagree, 2 = S omewhat disagree, 3 = Neutral, 4 = Somewhat agree, 5 = Strongly agree

**Table A1:** The Junior Students’ Internet Literacy Scale (JIL Scale).

Items	Scores				
1. We should respect different Internet cultures in the world.	1	2	3	4	5
2. I can use some Internet tools like office software and search engine.	1	2	3	4	5
3. I can show myself through social media such as WeChat, QQ, Douyin, and so on.	1	2	3	4	5
4. Keep a clear head and discern harmful information while surfing the Internet.	1	2	3	4	5
5. I realize that Internet has two sides.	1	2	3	4	5
6. If someone disagrees with me online, I will communicate with him sensibly instead of scolding him.	1	2	3	4	5
7. I can use what I have learned in the information technology class when I go online.	1	2	3	4	5
8. I can express my opinions and ideas through various media such as text, sound, images, etc.	1	2	3	4	5
9. When I study online, I am easily attracted by other irrelevant information.	1	2	3	4	5
10. I am good at using the Internet to expand my relationships.	1	2	3	4	5
11. I can access information via the web and sort out useful information to complete the learning tasks.	1	2	3	4	5
12. I can’t control how much time I spend on the Internet.	1	2	3	4	5
13. I can use the Internet to improve myself, such as learning meaningful knowledge or skills.	1	2	3	4	5
14. I can be immune to bad information while online.	1	2	3	4	5
15. I can use Internet resources creatively, such as searching for picture materials to make PowerPoint.	1	2	3	4	5
16. I don’t have a plan when I’m surfing the Internet.	1	2	3	4	5
17. I can find my favorite learning method online.	1	2	3	4	5
18. I can make new friends through the Internet.	1	2	3	4	5

## References

[1] China Internet Network Information Center (2021). The 47th China Statistical Report on Internet Development.

[2] China Internet Network Information Center (2020). 2019 China Research Report on Internet Usage by Minors.

[3] United Nations International Children’s Emergency Fund (2017). Children in a Digital World—The State of the World’s Children 2017.

[4] Katz I., Lemish D., Cohen R., Arden A. (2019). When parents are inconsistent: Parenting style and adolescents’ involvement in cyberbullying. J. Adolesc..

[5] Kuss D.J., Lopez-Fernandez O. (2016). Internet addiction and problematic Internet use: A systematic review of clinical research. World J. Psychiatry.

[6] Brand M., Young K.S., Laier C., Wolfling K., Potenza M.N. (2016). Integrating psychological and neurobiological considerations regarding the development and maintenance of specific Internet-use disorders: An Interaction of Person-Affect-Cognition-Execution (I-PACE) model. Neurosci. Biobehav. Rev..

[7] Boniel-Nissim M., Sasson H. (2018). Bullying victimization and poor relationships with parents as risk factors of problematic internet use in adolescence. Comput. Human Behav..

[8] McClure C.R. (1994). Network literacy: A role for libraries?. Inf. Technol. Libr..

[9] Association of College and Research Libraries (2000). Information Literacy Competency Standards for Higher Education.

[10] Livingstone S. (2008). Engaging with media—A matter of literacy?. Commun. Cult. Crit..

[11] Livingstone S. (2008). Internet Literacy: Young People’s Negotiation of New Online Opportunities.

[12] Leung L. (2010). Effects of Internet connectedness and information literacy on quality of life. Soc. Indic. Res..

[13] Lee S.J., Chae Y.G. (2012). Balancing participation and risks in children’s internet use: The role of internet literacy and parental mediation. Cyberpsychol. Behav. Soc. Netw..

[14] Len-Ríos M.E., Hughes H.E., McKee L.G., Young H.N. (2016). Early adolescents as publics: A national survey of teens with social media ac-counts, their media use preferences, parental mediation, and perceived Internet literacy. Public Relat. Rev..

[15] Yu L., Recker M., Chen S., Zhao N., Yang Q. (2018). The moderating effect of geographic area on the relationship between age, gender, and information and communication technology literacy and problematic internet use. Cyberpsychol. Behav. Soc. Netw..

[16] Durak H.Y., Saritepeci M. (2019). Modeling the effect of new media literacy levels and social media usage status on problematic internet usage behaviours among high school students. Educ. Inf. Technol..

[17] Leung L., Lee P.S.N. (2012). Impact of internet literacy, internet addiction symptoms, and internet activities on academic performance. Soc. Sci. Comput. Rev..

[18] Leung L., Lee P.S.N. (2012). The influences of information literacy, internet addiction and parenting styles on internet risks. New Media Soc..

[19] Stodt B., Wegmann E., Brand M. (2016). Predicting dysfunctional Internet use: The role of age, conscientiousness, and Internet literacy in Internet addiction and cyberbullying. Int. J. Cyber Behav. Psychol. Learn..

[20] Wegmann E., Stodt B., Brand M. (2015). Addictive use of social networking sites can be explained by the interaction of Internet use expectancies, Internet literacy, and psychopathological symptoms. J. Behav. Addict..

[21] Hatlevik O.E., Throndsen I., Loi M., Gudmundsdottir G.B. (2018). Students’ ICT self-efficacy and computer and information literacy: Determinants and relationships. Comput. Educ..

[22] Livingstone S., Helsper E. (2010). Balancing opportunities and risks in teenagers’ use of the internet: The role of online skills and internet self-efficacy. New Media Soc..

[23] Rohatgi A., Scherer R., Hatlevik O.E. (2016). The role of ICT self-efficacy for students’ ICT use and their achievement in a computer and information literacy test. Comput. Educ..

[24] Shapiro J.J., Hughes S.K. (1996). Information literacy as a liberal art?. Educom Rev..

[25] Li Q. (2012). The Empirical Research of Internet Literacy of the Middle School Student in Changchun. Master Thesis.

[26] Li B.M. (2012). Research on Children Internet Literacy. Ph.D. Thesis.

[27] Wu X.W., Na R., Li D. (2009). Research on the design of Network information literacy and competency scale for college students. Inf. Stud. Theory Appl..

[28] Bauer A.T., Ahooei E.M. (2018). Rearticulating Internet Literacy. J. Cyberspace Stud..

[29] Stodt B., Brand M., Sindermann C., Wegmann E., Li M., Zhou M., Sha P., Montag C. (2018). Investigating the effect of personality, internet literacy, and use expectancies in internet-use disorder: A comparative study between China and Germany. Int. J. Environ. Res. Public Health.

[30] Wu W.Y. (2017). Influence of College Students’ Internet Literacy on Internet Addiction. Ph.D. Thesis.

[31] Savolainen R. (2002). Network competence and information seeking on the Internet: From definitions towards a social cognitive model. J. Doc..

[32] Chen H.M., Yang X.M. (2004). The Internet literacy education of youth in the information age. J. Mass Commun. Mon..

[33] Li Z.Y. (2005). Cultivation mechanism and cultivation methods of web information literacy for the undergraduate. Inf. Sci..

[34] Bei J.H. (2006). An empirical study of college students’ Internet literacy. China Youth Study.

[35] Huang Y.Y. (2007). On the education of network media literacy for college students. J. Mass Commun. Mon..

[36] Ngulube P., Shezi M., Leach A. (2009). Exploring network literacy among students of St. Joseph’s Theological Institute in South Africa. S. Afr. J. Libr. Inf. Sci..

[37] Rheingold H. (2012). Net Smart: How to Thrive Online.

[38] Kim E., Yang S. (2016). Internet literacy and digital natives’ civic engagement: Internet skill literacy or Internet information literacy?. J. Youth Stud..

[39] Wang W.J., Liu H., Wang W., Dong R.C. (2021). Research on the evaluating indicators system of Internet literacy for K12 students. J. Cent. China Norm. Univ. Humanit. Soc. Sci..

[40] Young K.S. (1998). Caught in the Net: How to Recognize the Signs of Internet Addiction—And a Winning Strategy for Recovery.

[41] Eastin M.S., LaRose R. (2000). Internet self-efficacy and the psychology of the digital divide. J. Comput. Commun..

[42] Luo Z.H., Wan J.J., Liu Q.X., Fang X.Y. (2010). The relationship of Internet use, Internet special self-efficacy and Internet addiction in university students. Psychol. Dev. Educ..

[43] Currie C., Molcho M., Boyce W., Holstein B., Torsheim T., Richter M. (2008). Researching health inequalities in adolescents: The development of the Health Behaviour in School-Aged Children (HBSC) family affluence scale. Soc. Sci. Med..

[44] Byrne B.M. (2013). Structural Equation Modeling with Mplus: Basic Concepts, Applications, and Programming.

[45] Hu L., Bentler P.M. (1999). Cutoff criteria for fit indexes in covariance structure analysis: Conventional criteria versus new alternatives. Struct. Equ. Model. Multidiscip. J..

[46] Hair J.F., Black W.C., Babin B.J., Anderson R.E. (2010). Multivariate Data Analysis: A Global Perspective.

[47] Cattell R.B. (1966). The scree test for the number of factors. Multivar. Behav. Res..

[48] Kaiser H.F. (1960). The application of electronic computers to factor analysis. Educ. Psychol. Meas..

[49] Horn J.L. (1965). A rationale and test for the number of factors in factor analysis. Psychometrika.

[50] Malhotra N.K. (2006). Marketing Research: An Applied Orientation.

[51] Ford J.K., MacCallum R.C., Tait M. (1986). The application of exploratory factor analysis in applied psychology: A critical review and analysis. Pers. Psychol..

[52] Cronbach L.J. (1951). Coefficient alpha and the internal structure of tests. Psychometrika.

[53] Streiner D.L. (2003). Starting at the beginning: An introduction to coefficient alpha and internal consistency. J. Pers. Assess..

[54] Cicchetti D.V. (1994). Guidelines, criteria, and rules of thumb for evaluating normed and standardized assessment instruments in psychology. Psychol. Assess..

[55] Caplan S.E. (2010). Theory and measurement of generalized problematic Internet use: A two-step approach. Comput. Human Behav..

[56] Gámez-Guadix M., Villa-George F.I., Calvete E. (2012). Measurement and analysis of the cognitive-behavioral model of generalized problematic Internet use among Mexican adolescents. J. Adolesc..

[57] Valcke M., Bonte S., Wever B.D., Rots I. (2010). Internet parenting styles and the impact on Internet use of primary school children. Comput. Educ..

[58] Symons K., Ponnet K., Walrave M., Heirman W. (2017). A qualitative study into parental mediation of adolescents’ internet use. Comput. Human Behav..

[59] Soh P.C.H., Chew K.W., Koay K.Y., Ang P.H. (2018). Parents vs peers’ influence on teenagers’ Internet addiction and risky online activities. Telemat. Inform..

[60] Chou H.L., Chou C. (2019). A quantitative analysis of factors related to Taiwan teenagers’ smartphone addiction tendency using a random sample of parent-child dyads. Comput. Human Behav..

[61] Bandura A. (2009). Cultivate Self-Efficacy for Personal and Organizational Effectiveness.

[62] Wang J.L., Jackson L.A., Wang H.Z., Gaskin J. (2015). Predicting social networking site (SNS) use: Personality, attitudes, motivation and internet self-efficacy. Personal. Individ. Differ..

[63] Bandura A. (1997). Self-Efficacy: The Exercise of Control.

